# The Role of Integrin β1D Mislocalization in the Pathophysiology of Calpain 3-Related Limb–Girdle Muscular Dystrophy

**DOI:** 10.3390/cells14060446

**Published:** 2025-03-17

**Authors:** Andrea Valls, Cristina Ruiz-Roldán, Jenita Immanuel, Sonia Alonso-Martín, Eduard Gallardo, Roberto Fernández-Torrón, Mario Bonilla, Ana Lersundi, Aurelio Hernández-Laín, Cristina Domínguez-González, Juan Jesús Vílchez, Pablo Iruzubieta, Adolfo López de Munain, Amets Sáenz

**Affiliations:** 1 Neuromuscular Diseases Group, Neurosciences Area, Biogipuzkoa Health Research Institute, 20014 San Sebastian, Spain; 2 Center for Biomedical Network Research on Neurodegenerative Diseases (CIBERNED), Spanish Ministry of Science & Innovation, Carlos III Health Institute, 28029 Madrid, Spain; 3 Stem Cells and Aging Group, Bioengineering Area, Biogipuzkoa Health Research Institute, 20014 San Sebastian, Spain; 4 Neuromuscular Diseases Unit, Department of Neurology, Hospital de la Santa Creu i Sant Pau, 08041 Barcelona, Spain; 5 Institut de Recerca Sant Pau, IR-SantPau, 08041 Barcelona, Spain; 6 Center for Biomedical Network Research on Rare Diseases (CIBERER), Spanish Ministry of Science & Innovation, Carlos III Health Institute, 28029 Madrid, Spain; 7 Department of Neurology, Hospital Universitario Donostia, Osakidetza, 20014 San Sebastian, Spain; 8 Department of Traumatology, Donostialdea Integrated Health Organisation, Osakidetza, 20014 San Sebastian, Spain; 9 Department of Surgery, University of the Basque Country UPV/EHU, 20014 San Sebastian, Spain; 10 Department of Neuropathology, Hospital Universitario 12 de Octubre, 28041 Madrid, Spain; 11 Instituto de Investigación Sanitaria Hospital 12 de Octubre (imas12), 28041 Madrid, Spain; 12 Department of Pathology, Faculty of Medicine, Complutense University of Madrid (UCM), 28040 Madrid, Spain; 13 Neuromuscular Unit, Department of Neurology, Hospital 12 de Octubre, 28041 Madrid, Spain; 14 Neuromuscular and Ataxias Research Group, Instituto de Investigación Sanitaria La Fe, 46026 Valencia, Spain; 15 Neuromuscular Diseases Unit, Neurology Department, Hospital Universitari I Politècnic La Fe, 46026 Valencia, Spain; 16 Neurogenetics, RNA Biology and Therapies Group, Neurosciences Area, Biogipuzkoa Health Research Institute, 20014 San Sebastian, Spain; 17 Department of Neurology and Neurosurgery, Montreal Neurological Hospital and Institute, McGill University, Montreal, QC H3A 2B4, Canada; 18 Department of Neurosciences, University of the Basque Country UPV-EHU, 20014 San Sebastian, Spain; 19 Faculty of Medicine, University of Deusto, 48007 Bilbao, Spain

**Keywords:** limb-girdle muscular dystrophy, LGMDR1, calpain 3, integrin β1, costamere

## Abstract

Limb–girdle muscular dystrophy R1 (LGMDR1) is characterized by progressive proximal muscle weakness due to mutations in the *CAPN3* gene. Little is known about CAPN3’s function in muscle, but its loss results in aberrant sarcomere formation. Human muscle structure was analyzed in this study, with observations including integrin β1D isoform (ITGβ1D) mislocalization, a lack of Talin-1 (TLN1) in the sarcolemma and the irregular expression of focal adhesion kinase (FAK) in LGMDR1 muscles, suggesting a lack of integrin activation with an altered sarcolemma, extracellular matrix (ECM) assembly and signaling pathway deregulation, which may cause frailty in LGMDR1 muscle fibers. Additionally, altered nuclear morphology, centrosome distribution and microtubule organization have been found in muscle cells derived from LGMDR1 patients.

## 1. Introduction

Limb-girdle muscular dystrophy R1-calpain 3 related is one of the most common autosomal recessive limb–girdle muscular dystrophies. It is characterized by progressive proximal muscle weakness. For most patients, onset occurs in adolescence, and patients usually lose independent ambulation after around 20 years of progression [1,2].

Although CAPN3, a muscle-specific protease, was first described in 1989 [3], its function in muscle is still not completely understood. However, it has been reported that a lack of CAPN3 alters several structures and signaling pathways in the skeletal muscle. This protein binds to titin, a gigantic protein that spans from the M- to Z-lines of the muscle sarcomere [4,5]. The loss of CAPN3 results in aberrant sarcomere formation [6] and alterations in the expression of several genes in LGMDR1 patients’ muscles [7].

The costamere is a morphological structure in striated muscles aligned with myofibril Z-discs [8]. It may participate in the assembly and stabilization of sarcomeres [8,9,10]. Costamere components showed altered expression in LGMDR1 patients at both the gene level, like in *ITGB1BP2* and *ANOS1*, and the protein level, like in melusin or the integrin β1D isoform (ITGβ1D) [7,11].

Another important cellular component is the centrosome, a non-membranous organelle composed of two centrioles. The centrosome is the main microtubule-organizing center (MTOC) in proliferating cells [12]. Moreover, the centrosome contains a pair of centrioles where the microtubule minus ends anchor [13,14]. Specifically, in muscle cells, the centrosomes are distributed with a perinuclear localization [15,16]. Notably, a perinuclear distribution of CAPN3 has been suggested [17], and Winter and colleagues [18] showed that it is a functional constituent of the centrosomes in mice myoblasts. Furthermore, ITGβ1D regulates the integrity of the centrosome [19].

Centrioles are crucial for the formation of the primary cilia, that is, microtubule-based sensory organelles required for signal transduction during development and adult tissue homeostasis [20,21,22]. To control cellular functions, changes in the extracellular environment are detected by the cilia, and they transmit signaling information to the cell. Hedgehog, Wnt, Notch and mTOR signaling pathways are among the principal signaling pathways coordinated by the primary cilia that regulate developmental processes and organ functions [23,24,25,26]. These signaling pathways are required for correct cellular homeostasis, yet the Wnt and mTOR signaling pathways appear altered in LGMDR1 patients [27].

On the other hand, the nucleus shows a tight connection to the centrosome [20]. Ding and colleagues [28] showed that CAPN3 is recruited by Def (Digestive organ expansion factor) into the nucleolus to form the Def-CAPN3 complex. Although the nucleus has not been analyzed in depth in LGMDR1, CAPN3, which contains a nuclear translocation signal within its amino acid sequence [3], has been suggested to be implicated in nuclear function [17,18,29].

In this work, we report the mislocalization of costamere proteins in LGMDR1 myofibers, swollen vascular vessels in the LGMDR1 muscle and a dispersed distribution of Talin-1 in muscle cells and their nuclei. Furthermore, we describe, for the first time, the altered morphology of nuclei and nucleoli, as well as clusters of centrosomes and aberrant mitotic spindles in LGMDR1 muscle cells. As ITGβ1D is common among all these pathological features, we propose that it takes the spotlight in future therapeutic strategies.

## 2. Materials and Methods

The LGMDR1 muscle samples used in this study came from our historical patients’ series. All patients presented two mutations in the *CAPN3* gene (Table 1 and Table 2). Healthy patients who had surgery for bone fractures provided the control muscles, and muscle biopsies were obtained during the surgery. The Ethics Committee on the Use of Human Subjects in Research at Donostia University Hospital authorized the forms that were used to seek informed consent from each participant, who signed voluntarily prior to the collection of muscle samples.

### 2.1. Primary Human Skeletal Muscle Culture

Human muscle samples were cultured in a monolayer as previously described (Askanas 1975 [30]). Myoblasts and non-myogenic cells were purified by sorting primary cultures through immunomagnetic selection based on the early cell surface marker CD56 (Miltenyi Biotec, Bergisch, Gladbach, Germany). Myoblasts (CD56+) and non-myogenic cells (CD56−) were seeded at 2500–3000 cells/cm^2^ in proliferation medium. This medium contained 10% FBS (Gibco, Thermo Fisher Scientific, Waltham, MA, USA), DMEM (Gibco, Thermo Fisher Scientific, Waltham, MA, USA), M-199 (Lonza Group Ltd., Basilea, Switzerland), insulin (Sigma-Aldrich, San Luis, MO, USA), L-Glutamine (Gibco, Thermo Fisher Scientific, Waltham, MA, USA), penicillin/streptomycin (Gibco, Thermo Fisher Scientific, Waltham, MA, USA) and growth factors (PeproTech, Cranbury, NJ, USA). Myotubes were obtained through the replacement of the proliferation medium in CD56+ 100% confluent cultures, and from one culture containing 2% FBS without growth factors. Myotube cultures were maintained for 15 days. The medium was changed every two days during the culturing process.

### 2.2. Immunofluorescence of Muscle Sections

The detection of costamere proteins was performed in 7 µm skeletal muscle cryosections. Tissue fixation was performed using 4% PFA (Electron Microscopy Sciences, Hatfield, PA, USA) for 5 min at room temperature (RT). Incubation for 6 min with pure frozen methanol (−20 °C) was used to permeabilize muscle sections. Then, blocking solution [5% BSA IgGfree (Leica Biosystems Nussloch GmbH, Nussloch, Germany), 2% goat serum (Abcam plc, Cambridge, UK), 0.025% Tween20 (Sigma-Aldrich, San Luis, MO, USA)] was added for 2 h at RT. Primary antibody incubation was performed overnight at 4 °C at the indicated dilutions (Table S1—Supplementary), and 1:400 diluted fluorescent secondary antibodies were added at RT for 2 h, protected from light (Table S2—Supplementary). The incubation of 1:30 diluted anti-dystrophin (Santa Cruz Biotechnology, Dallas, TX, USA) or laminin (Novus Biologicals, Centennial, CO, USA) labeled with Alexa Fluor 647 for 1 h under light-protected conditions was used to delimitate the subsarcolemmal region of the muscle fibers. Nuclei were counterstained with Hoechst (Thermo Fisher Scientific, Waltham, MA, USA).

To determine the vascular endothelium association of the costamere proteins, as well as the number of blood vessels, the aforementioned protocol was followed, with the ulex-biotin marker (Vector Laboratories, Newark, CA, USA) added to the primary antibody solution in a 1:333 dilution. Afterwards, avidin-AF488 (Invitrogen, Waltham, MA, USA) was added to the secondary antibody solution in a 1:2000 dilution.

### 2.3. Immunofluorescence of Cell Cultures

CD56− cells and myoblasts were cultured in an optical µ-Plate 96 Well 3D (Ibidi GmbH, Gräfelfing, Germany) and myotubes in an optical µ-Plate 96 Well Square (Ibidi GmbH, Gräfelfing, Germany) coated with 0.5% and 0.1% gelatin (Sigma-Aldrich, San Luis, MO, USA), respectively. Cells were fixed with 4% PFA (Electron Microscopy Sciences, Hatfield, PA, USA) for 5 min at RT. After several PBS washes, the cells were permeabilized and blocked with blocking solution [0.3% Triton-X (Sigma-Aldrich, San Luis, MO, USA) and 5% goat serum (Abcam) in PBS] for 2 h at RT. Primary antibodies were diluted in blocking buffer and incubated overnight at 4 °C (Table S1—Supplementary Materials). Well plates were washed with a washing solution [0.025% Tween 20 (Sigma-Aldrich, San Luis, MO, USA) in PBS], and fluorescent secondary antibodies were added and incubated for 2 h at RT (Table S2—Supplementary). Hoechst (Thermo Fisher Scientific, Waltham, MA, USA) was used to stain the nuclear regions of cells by incubating cells for 3 min at RT. After several washes with the washing solution, a storage solution [0.1% sodium azide (Sigma-Aldrich, San Luis, MO, USA) in PBS] was added to preserve the cells until microscopy analysis.

Here, specific primary antibodies were used to detect cellular structures. Anti-γ-tubulin antibody was used to detect the centrosome location/structure, since this protein is enriched in centrosomes [31,32]. The study of the primary cilia was performed after immunodetection with anti-Arl13b, an abundant protein in this cell structure [33] (Table S1—Supplementary Materials).

### 2.4. Wound-Healing (WH) Assay in CD56− Cells

Cells were seeded in an optical µ-Slide 8 Well (Ibidi GmbH, Gräfelfing, Germany) coated with 0.5% gelatin (Sigma-Aldrich, San Luis, MO, USA) until confluence was reached. A wound was made using a 200 µL pipette tip, and cells were fixed with 4% PFA (Electron Microscopy Sciences, Hatfield, PA, USA) 6 h later. Centrosome orientation analysis was performed after immunostaining with α- and γ-tubulin antibodies, as described above (Table S1—Supplementary Materials).

### 2.5. Confocal Microscopy Analysis and Statistics

Images of the muscle sections and cells were digitally captured with a confocal microscope LSM900 (Carl Zeiss AG, Oberkochen, Germany) and ZenBlue3.0 software. The processing or analysis of these images was performed using Image J (v1.53t).

- Protein quantification of costamere proteins in the sarcoplasm of muscle fibers: Fiber segmentation and quantification of the fluorescence intensity (average fluorescence intensity within the fiber) corresponding with a direct proportion of protein expression was performed. Different amounts of the fibers were analyzed for each protein: 150–240 fibers per control/patient group for ITGβ1D analysis; 65–200 fibers per control/patient group for TLN1; 40–80 fibers per control/patient group for vinculin (VCL); 140–400 fibers per control/patient group for focal adhesion kinase (FAK); and 55–160 fibers per group for integrin-linked kinase (ILK). The statistical significance was assessed using an unpaired *t*-test after the elimination of outliers by the Rout method (Q = 1%).
- Blood vessel quantification in skeletal muscle: The blood vessels of 450–890 fibers (after fiber segmentation) were analyzed per group.
- Nuclear morphology analysis in cells and skeletal muscle tissue sections: Nucleus segmentation and shape descriptor (circularity, roundness, aspect ratio, area and perimeter) analyses were performed. Overall, 800–1000 nuclei were analyzed per group for CD56− cells, 700–1000 nuclei per group for myoblasts, 180–200 nuclei for myotubes and 500–700 nuclei for skeletal muscle sections. Only laminin-delimitated myonuclei were considered for the analysis of skeletal muscle nuclei. Statistical significance was assessed using an unpaired *t*-test after the elimination of outliers via the Rout method (Q = 1%).
- Centrosome–nucleus distance in skeletal muscle cells: The centrosome–nucleus distance was assessed using antibodies against ϒ-tubulin. Overall, 100 CD56− cells and 150 myoblasts were analyzed per group. The statistical significance was assessed using an unpaired *t*-test after the elimination of outliers via the Rout method (Q = 1%).
- Quantification of cytoskeletal α-tubulin in muscle cells: α-tubulin fluorescence intensity was normalized to the cell number. For CD56− cells, 250 control and 500 patient cells were assessed; for myoblasts, there were 500 control and 1200 patient cells. The statistical significance was assessed using an unpaired *t*-test after the elimination of outliers via the Rout method (Q = 1%).
- Centrosome positioning assessment in CD56− cells: The number of cells with a misoriented centrosome after a WH assay was assessed. The statistical significance was assessed using an unpaired *t*-test.
- Quantification of ciliated myoblasts: The number of ciliated cells was measured. The statistical significance was assessed using an unpaired *t*-test.

## 3. Results

### 3.1. Costamere Proteins Are Fiber-Type-Dependent and Also Present in the Endothelial Tissue

**Figure 1:**
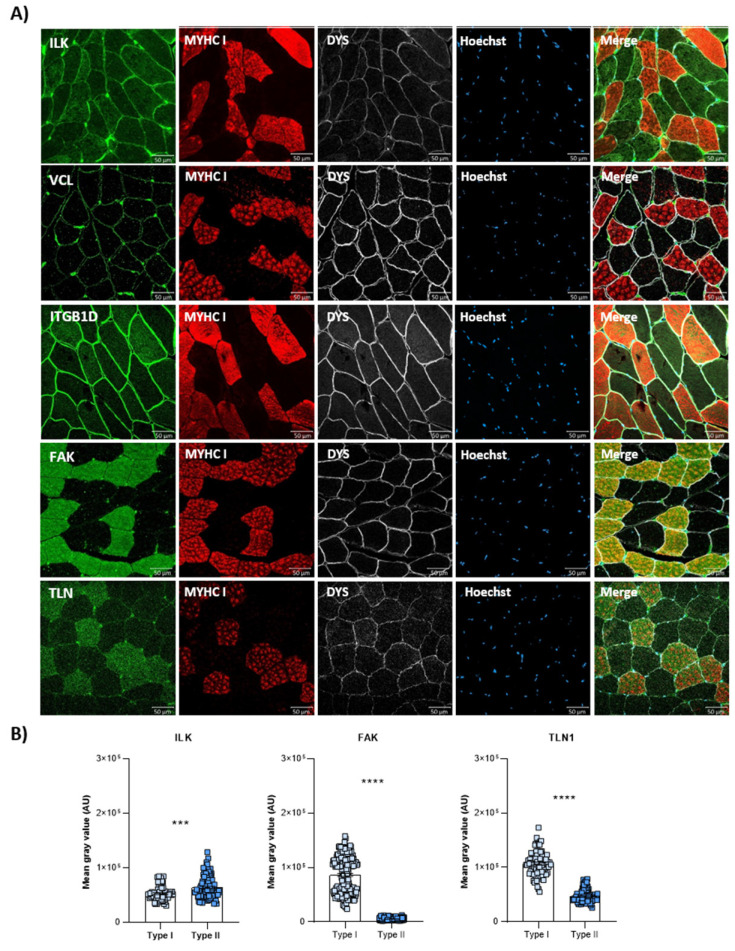
Costamere protein distributions in skeletal muscle sections of healthy control muscles. (**A**) Immunofluorescence representative images of ILK, VCL, ITGβ1D, FAK and TLN1 (green), MYHC I (red) and DYS (white) in control muscle fibers. Hoechst (blue) was used as a nuclear marker. Scale bars: 50 μm. (**B**) Fluorescence intensity quantification of the costamere proteins (n = 2). Error bars represent the standard error of the mean (SEM). *** *p* < 0.001, **** *p* < 0.0001.

For a better understanding of the distribution of costamere proteins in healthy skeletal muscle, we studied ILK, VCL, ITGB1D, FAK, TLN1 and α- and β-parvin due to their established role in costamere formation and/or maintenance [8,34,35,36,37,38]. ILK, VCL, ITGβ1D and TLN1 were localized at the sarcolemma and sarcoplasm of the muscle fiber. FAK was the only protein that did not show sarcolemmal expression (Figure 1A). A protein expression analysis of ILK, TLN1 and FAK, which showed clear sarcoplasmic distribution, also demonstrated fiber-type specific expression (Figure 1). A higher sarcoplasmic expression of TLN1 and FAK proteins was observed in type I or slow fibers. By contrast, ILK showed predominantly sarcoplasmic expression in type II or fast fibers (Figure 1).

**Figure 2:**
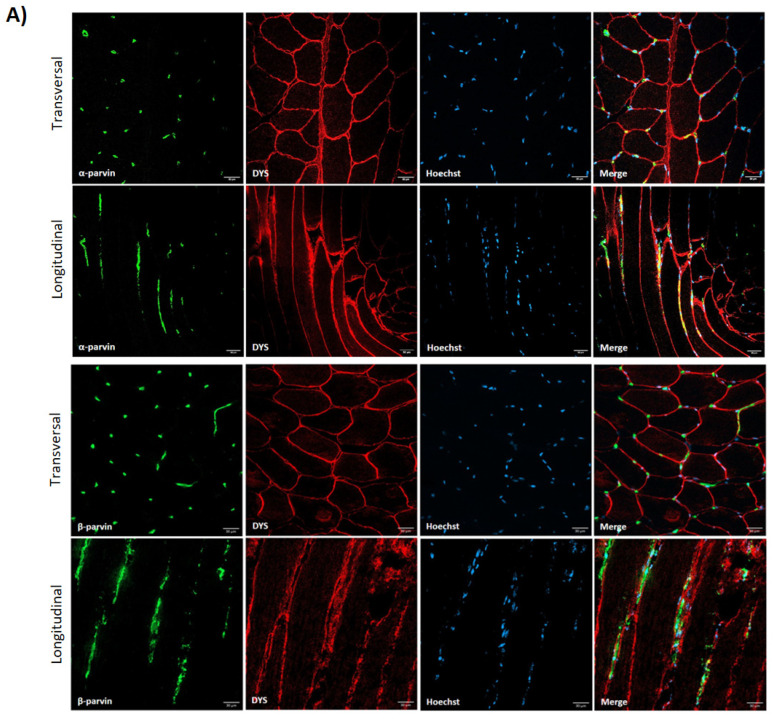

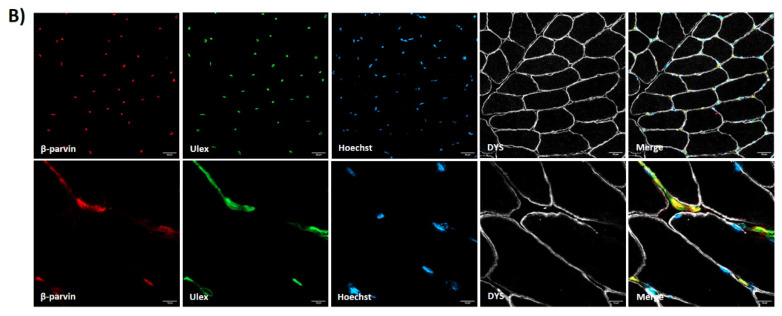
The distribution of α- and β-parvins in the muscle fiber (**A**) and the vascular endothelial distribution of β-parvin (**B**) in healthy control muscles. Ulex was used as a vascular endothelium marker. Scale bars: 30 μm (**A**) and (**B upper panel**) and 10 μm (**B lower panel**). (**A**) α- and β-parvins (green), dystrophin (red), Hoechst (blue). (**B**) β-parvin (red), ulex (green), Hoechst (blue), dystrophin (white).

**Figure 3:**
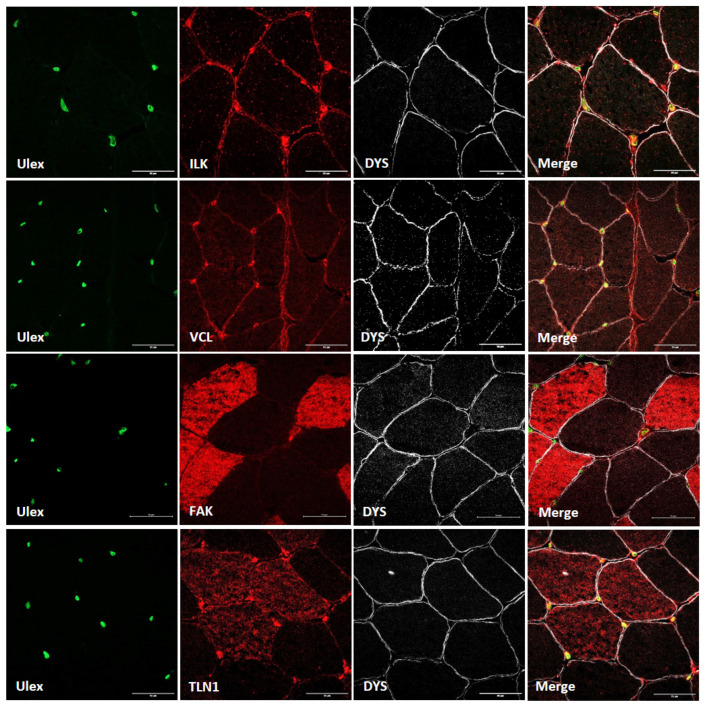
Ulex and costamere protein co-staining in healthy control muscle. Scale bars: 50 µm. Ulex (green); ILK, VCL, FAK and TLN1 (red); dystrophin (white).

Contrary to previous proteins, α-parvin and β-parvin showed localization close to the blood vessels in muscle tissue, with no expression in other regions of the muscle fiber (Figure 2). Therefore, after double-staining with β-parvin and ulex, a vascular endothelial marker, the expression of these proteins in the blood vessels of muscle tissue was confirmed (Figure 2). Finally, as ILK, TLN1, FAK and VCL were also expressed outside the muscle fiber, double-staining was performed with ulex, and the vascular endothelial expression of these proteins was also confirmed (Figure 3).

### 3.2. Abnormal Distribution of ITGβ1D and TLN1 in LGMDR1 Patients’ Muscle

**Figure 4:**
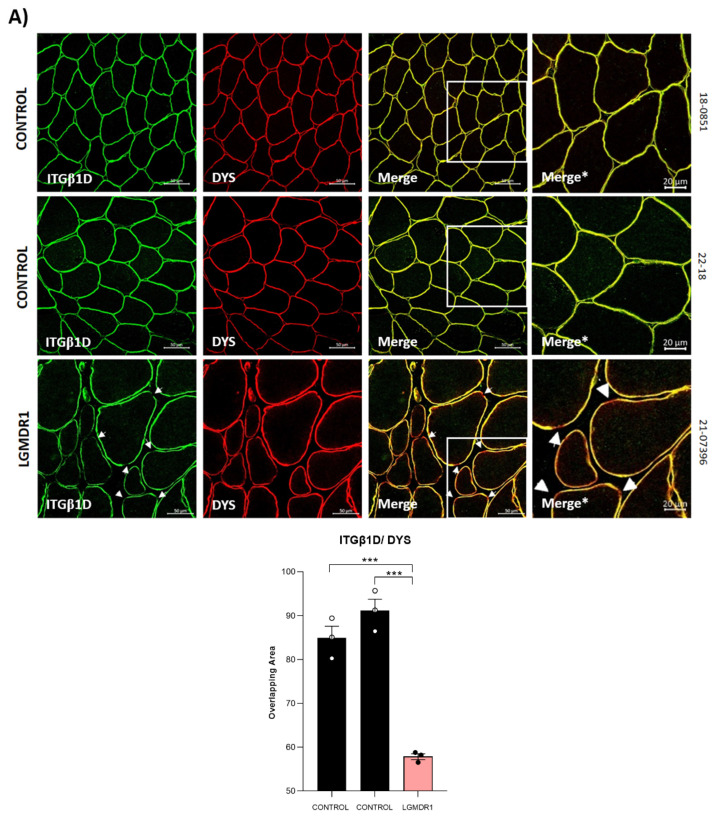

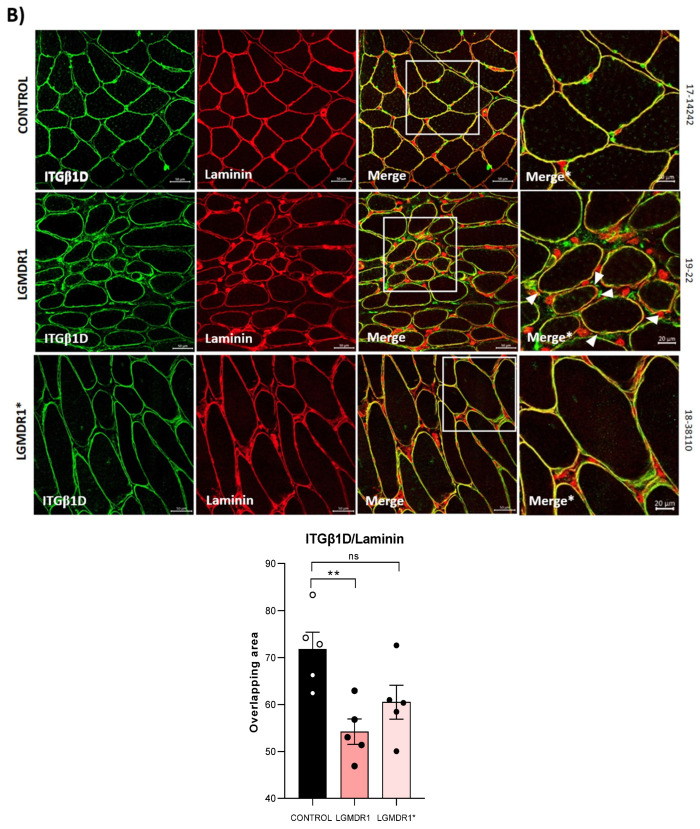
The sarcolemmal distribution of ITGβ1D in the muscle. (**A**) Dystrophin as a sarcolemmal marker (red) and the quantification of co-localization between ITGβ1D and DYS in the muscle of the two controls and a LGMDR1 patient. Error bars represent the standard error of the mean (SEM). *** *p* < 0.001. (**B**) Laminin as a sarcolemmal marker (red) and the quantification of co-localization between ITGβ1D and laminin in the muscle of one control and two LGMDR1 patients. Error bars represent the standard error of the mean (SEM). ** *p* < 0.01. White arrows indicate areas without ITGβ1D expression. Scale bars: 50 µm. Merge*, higher magnification of the white squares. Scale bars: 20 µm. ITGβ1D (green), laminin and DYS (red). ns: not significant.

**Figure 5:**
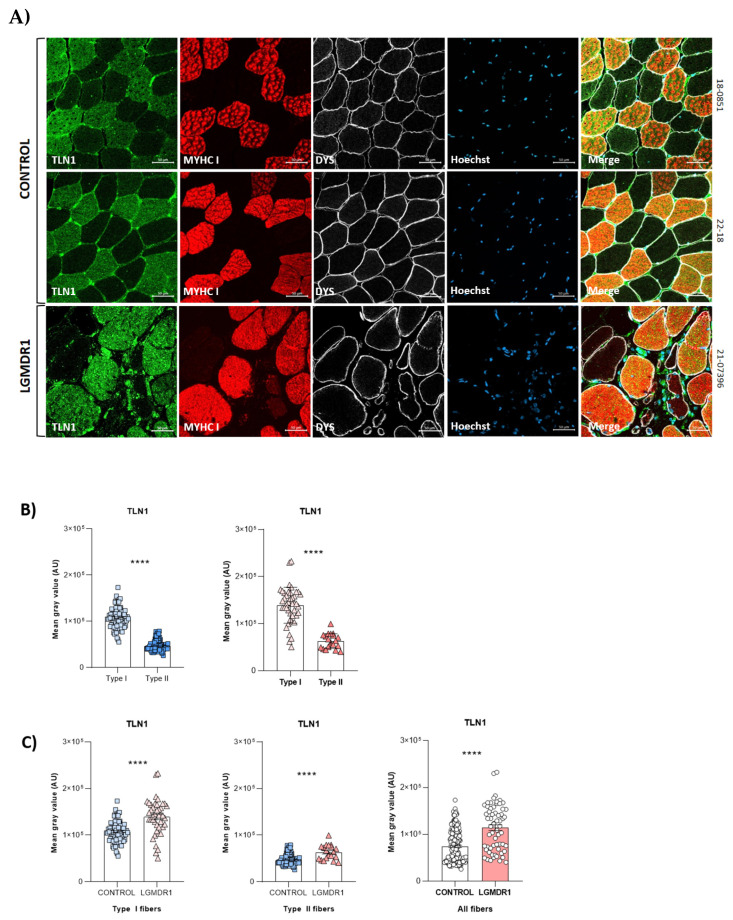
(**A**,**B**) TLN1 distribution (representative images) and quantification according to fiber type in two human control muscle samples and a LGMDR1 patient sample. (**C**) TLN1 expression comparing LGMDR1 patients and controls in different fiber types. Error bars represent the standard error of the mean (SEM). **** *p* < 0.0001. Scale bars: 50 μm.

**Figure 6:**
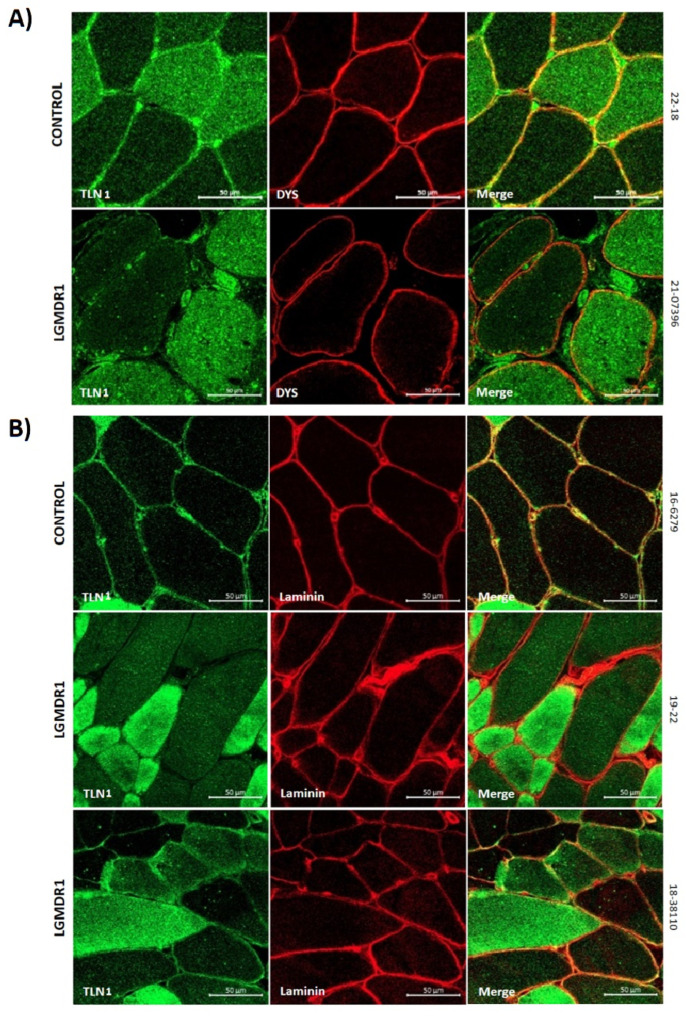
The distribution of TLN1 in control (n = 2) and patients’ (n = 3) muscle fibers. (**A**) TLN1 (green) and DYS (red) co-staining in muscles from one healthy control and one LGMDR1 patient. (**B**) TLN1 (green) and laminin (red) co-staining in muscles from one healthy control and two LGMDR1 patients (18-38110, LGMDR1 patient with a very mild phenotype). Scale bars: 50 µm.

**Figure 7:**
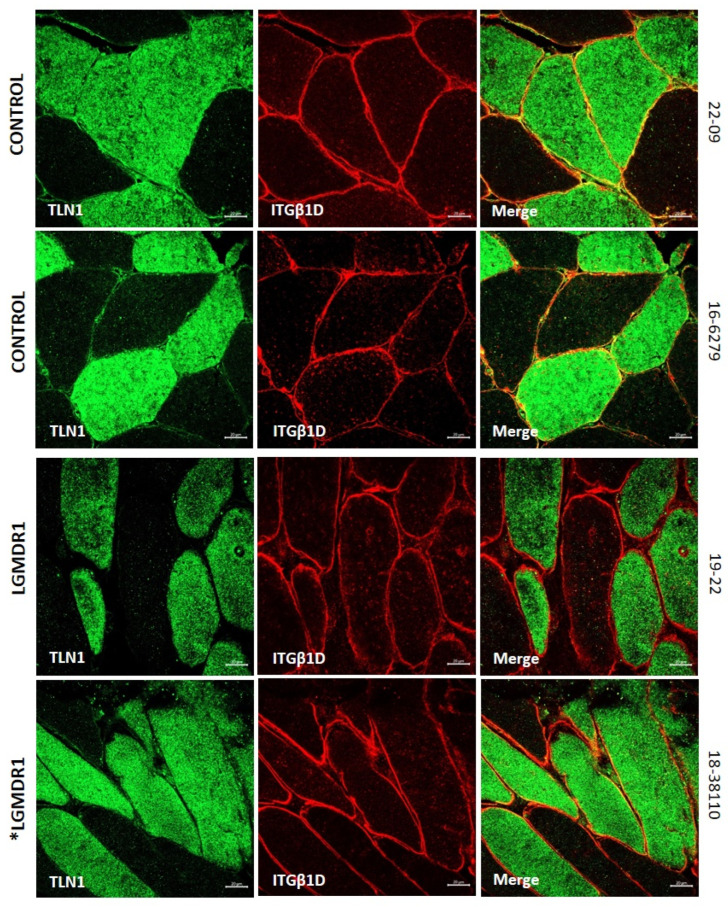
TLN1-ITGβ1D co-localization in muscle fibers. TLN1 (green), ITGβ1D (red) co-staining in the skeletal muscle fibers of two LGMDR1 patients (*LGMDR1 patient with a very mild phenotype) and two healthy controls. White arrowheads indicate co-localization of TLN1 with ITGβ1D. Scale bars: 20 µm.

We demonstrated that ITGβ1D is expressed in both the sarcolemma and cytoplasm in control muscles. However, the expression of ITGβ1D in the LGMDR1 patient’s sarcolemma is patchy, with discontinuous labeling using dystrophin as an inner sarcolemmal marker (Figure 4A). The observed pattern was confirmed, using laminin as an outer sarcolemmal marker, which interacts with integrin β1 in the ECM (Figure 4B). TLN1, which was preferentially expressed in type I fibers in the control and patients, was highly expressed in the cytoplasm of the patients’ fibers. Moreover, while its localization in the sarcolemma was clear in the control muscle fibers, it was absent in the sarcolemma of the patients’ muscle fibers (Figure 5 and Figure 6). Accordingly, when ITGβ1D and TLN1 were analyzed together, they showed clear co-localization in control muscles that was not observed in the LGMDR1 patients’ muscles (Figure 7). Patient 18-38110, who had a strikingly benign phenotype, presented good muscle structure. Accordingly, almost normal ITGβ1D and TLN1 distributions were observed with the co-localization of these proteins in some areas of the sarcolemma, thus, confirming a correlation between disease progression and the co-localization/interaction of ITGβ1D and TLN1 (Figure 6 and Figure 7).

**Figure 8:**
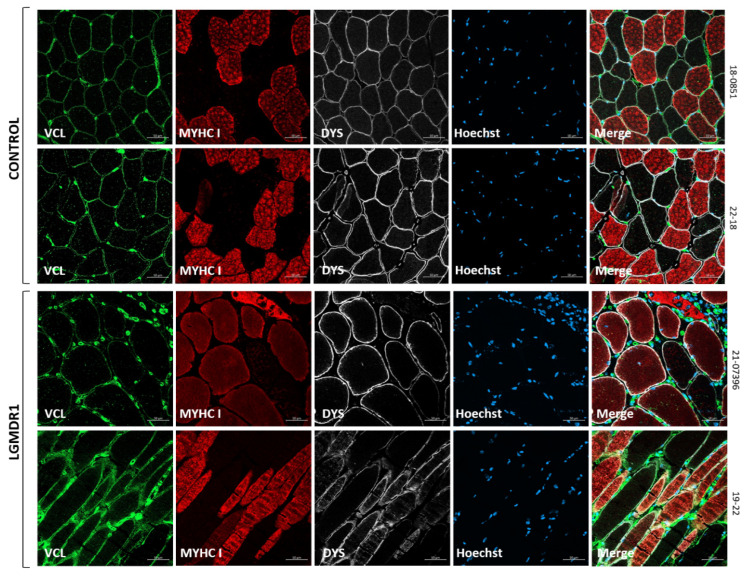
VCL distribution in two human control and two LGMDR1 patients’ muscles. VCL (green), MYHC I (red), DYS (white), Hoechst (blue). Scale bars: 50 μm.

We next verified the expression of VCL, which did not show preferential expression in any fiber type in the control or patients; it showed more expression in the blood vessels in LGMDR1 muscle fibers (Figure 8).

**Figure 9:**
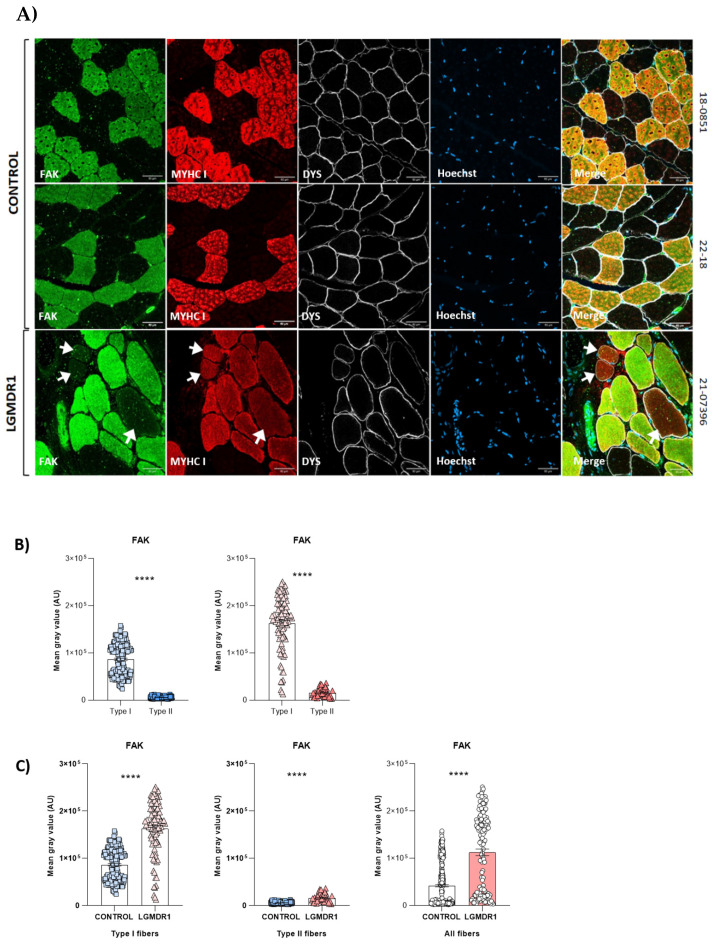
FAK distribution in muscle fibers. (**A**) FAK distribution in two human control muscles and a LGMDR1 patient’s muscle. White arrows indicate type I fibers that do not express FAK in one of the LGMDR1 patient. (**B**) FAK quantification according to fiber type. (**C**) Comparing the FAK expression between a LGMDR1 patient and controls in different fiber types. Error bars represent the standard error of the mean (SEM). **** *p* < 0.0001. Scale bars: 50 μm.

**Figure 10:**
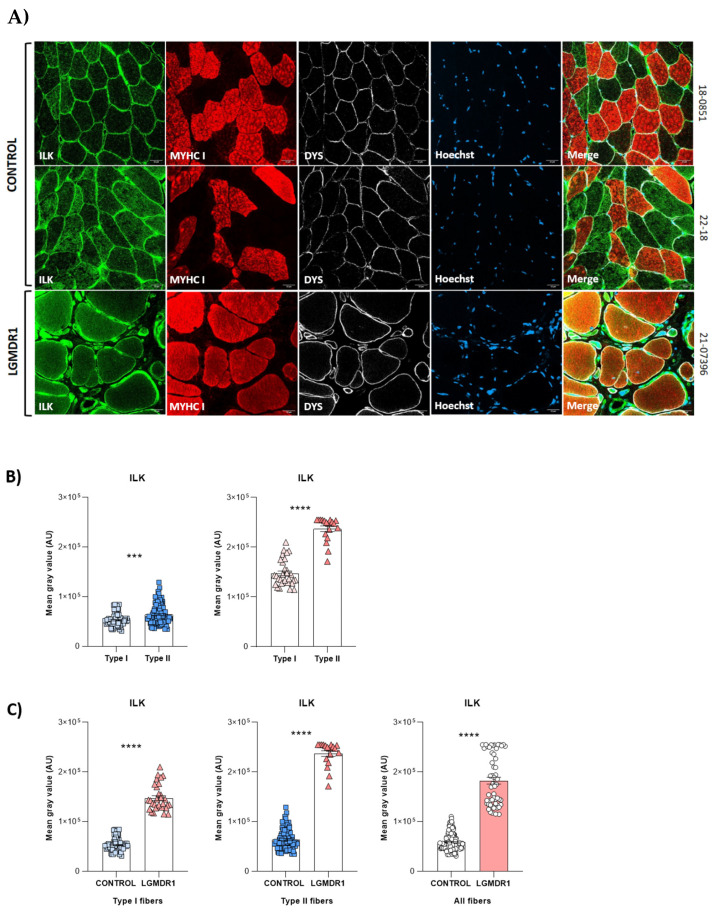
(**A**,**B**) ILK distribution and quantification according to fiber type in two human control muscles and one LGMDR1 patient’s muscle. (**C**) Comparing the ILK expression in a LGMDR1 patient and the controls in different fiber types. Error bars represent the standard error of the mean (SEM). *** *p* < 0.001, **** *p* < 0.0001. Scale bars: 50 μm.

**Figure 11:**
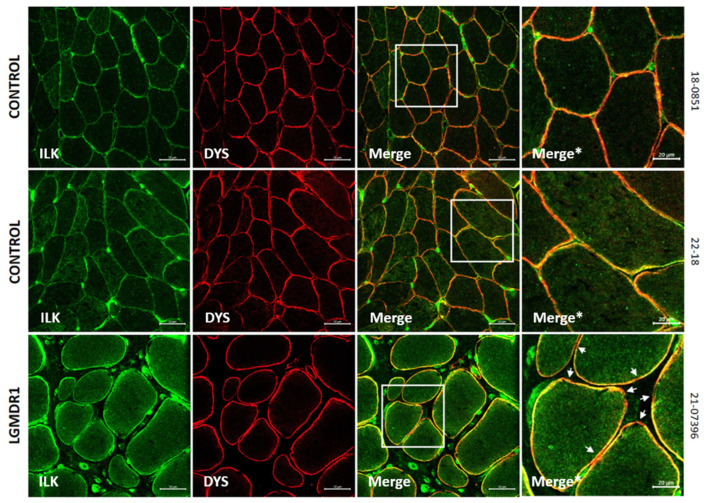
ILK expression in control and LGMDR1 skeletal muscle fibers. A predominance of dystrophin signal is observed in the sarcolemma in the controls, but in the patient, a greater amount of ILK is observed. White arrows indicate the only regions without ILK expression. Scale bars: 50 μm. In magnifications, scale bars: 20 μm. Merge*, higher magnification of the white squares.

On the other hand, FAK localized predominantly to type I fibers in both controls and patients, but its expression was consistently higher in the LGMDR1 patients’ samples (Figure 9). Furthermore, some type I fibers showed low FAK expression in LGMDR1 muscles (Figure 9A). Finally, ILK appeared to be predominantly expressed in type II fibers in both control and LGMDR1 muscles (Figure 1 and Figure 10). Specifically, its sarcoplasmic and sarcolemmal expression were significantly higher in both types of muscle fibers from the LGMDR1 patient compared to the healthy controls (Figure 10 and Figure 11). Finally, the co-localization of ILK and dystrophin was observed, but a higher level of expression of the dystrophin itself was clear in the controls.

### 3.3. Abnormal Blood Vessel Morphology in LGMDR1 Patients’ Muscles

Muscle function is strictly related to the vascular composition of skeletal muscle fibers [39]. Since costamere proteins are also expressed in smooth muscle, their expression and morphology in skeletal muscle fibers were analyzed.

**Figure 12:**
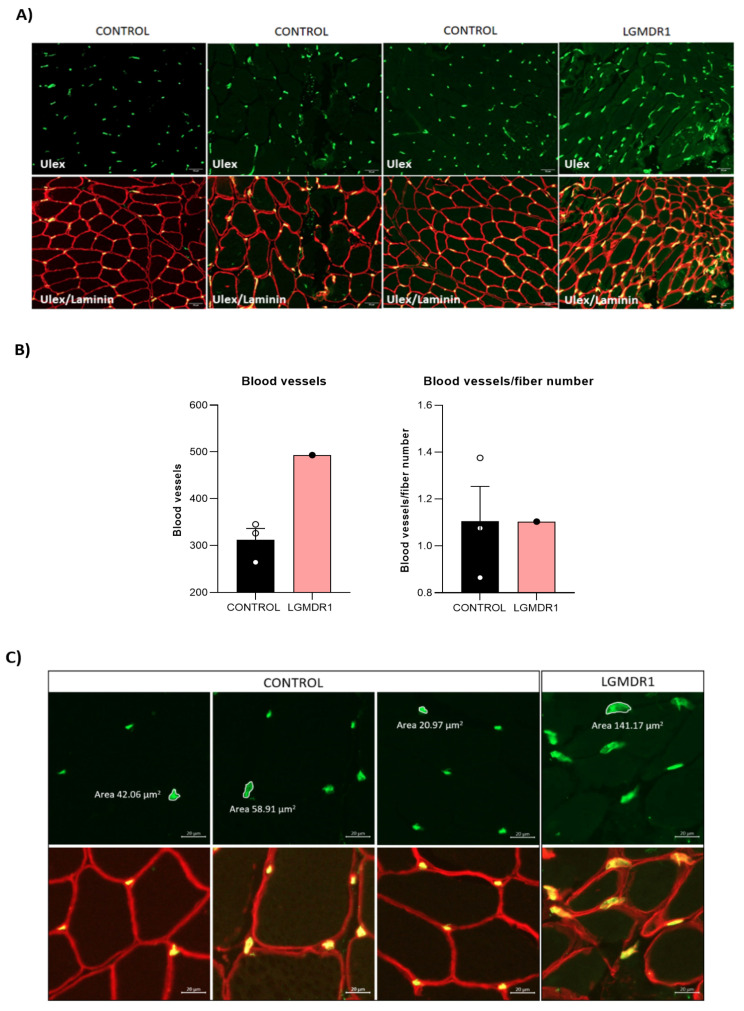
Vascular endothelium quantification and morphology in skeletal muscle fibers. (**A**) Representative images of the blood vessels in skeletal muscle (3 controls and 1 patient). Ulex (green) and laminin (red). Scale bars: 20 μm. (**B**) Blood vessel quantification, normalized by number of fibers. Error bars represent the standard error of the mean (SEM). (**C**) Abnormal and swollen blood vessel morphology in LGMDR1 patient’s muscle. Ulex (green), laminin (red). Scale bars: 10 μm.

The quantification of blood vessels showed a higher number of vessels in the LGMDR1 patients. When the number of blood vessels was normalized to the number of muscle fibers, this difference disappeared. Moreover, an abnormal and swollen morphology of the vessels was observed in LGMDR1 patients’ muscles (Figure 12).

### 3.4. TLN1 Localizes in the Nucleolus in CD56− Cells, Myoblasts, Myotubes and Muscle

**Figure 13:**
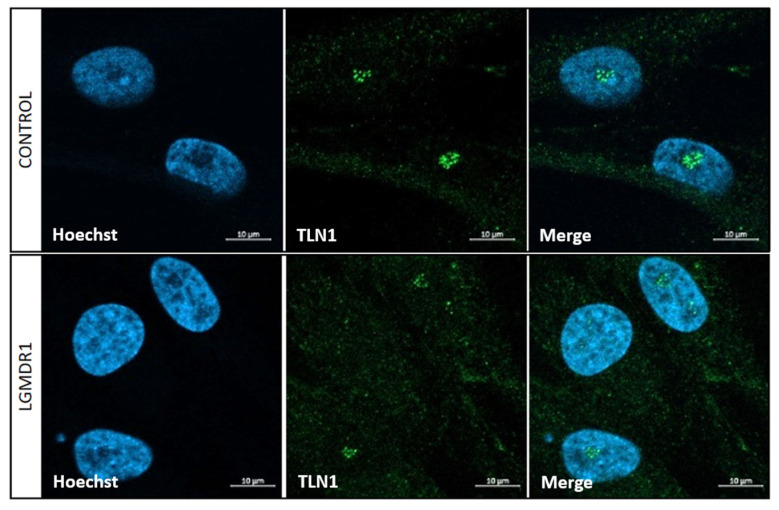
Nuclear localization of TLN1 in CD56− cells from controls and LGMDR1 patients. TLN1 distribution in the nucleus of CD56− cells. TLN1 (green), Hoechst (blue). Scale bars: 10 μm.

**Figure 14:**
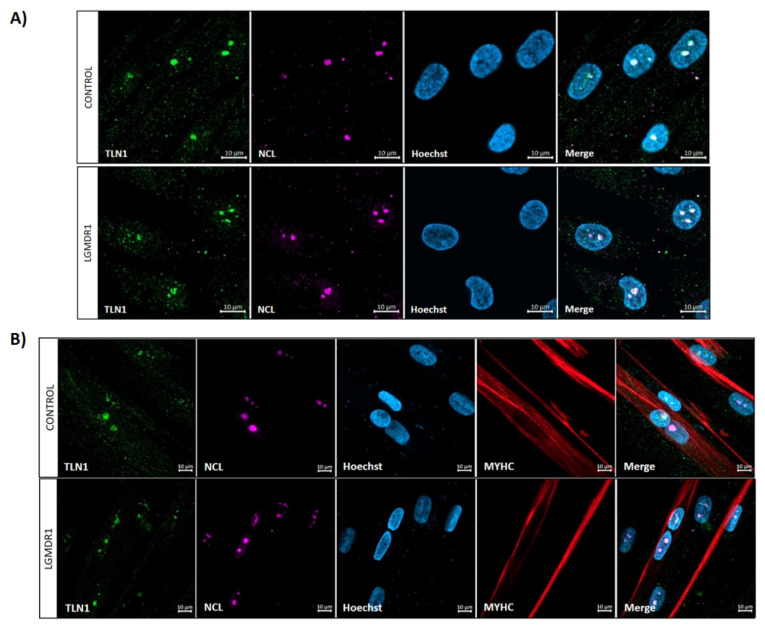
Nucleolar TLN1 localization in LGMDR1 and control (**A**) myoblasts and (**B**) myotubes. TLN1 (green), NCL (pink), Hoechst (blue), MYHC (red). Scale bars: 10 μm.

**Figure 15:**
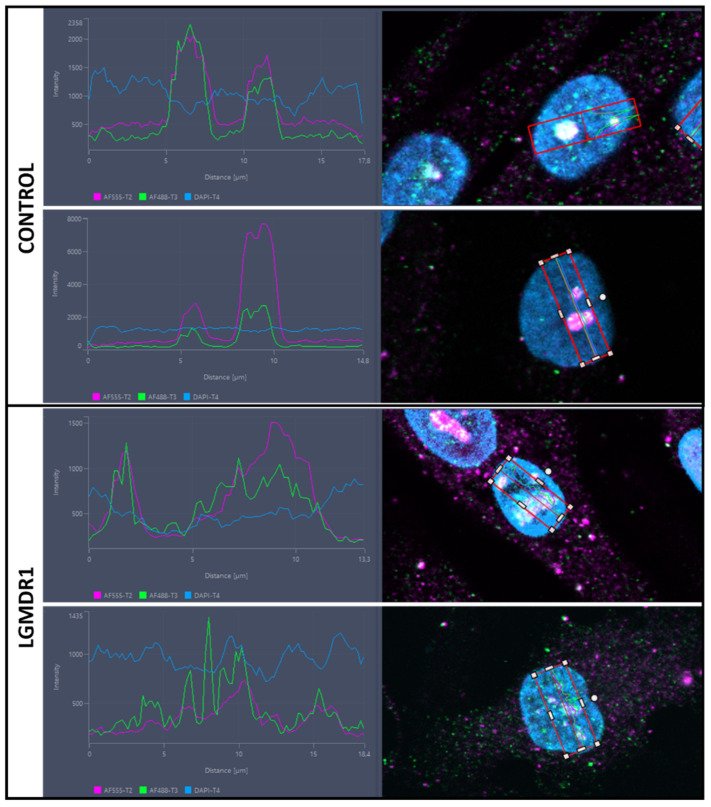
TLN1 and NCL dispersion throughout the nuclei of myoblasts from LGMDR1 patients. Plotted distance in µm (*x* axis), fluorescence intensity (*y* axis). TLN1 (green), NCL (pink), Hoechst (blue).

**Figure 16:**
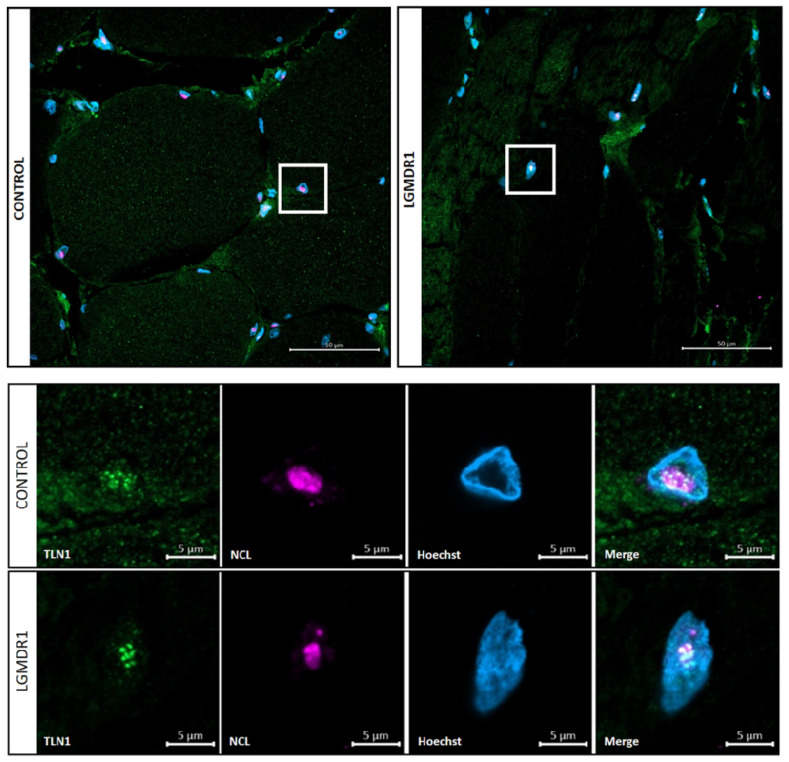
Nucleolar TLN1 localization in LGMDR1 and control skeletal muscle fibers. Amplification of the white squares is shown in the lower panel. Scale bars: 50 (**upper panel**) and 5 (**lower panel**) μm. TLN1 (green), NCL (pink), Hoechst (blue).

After tissue analysis, we proceeded to analyze primary cultured cells from muscle biopsies. Specifically, TLN1 appeared to be expressed in the nucleus of the non-myogenic CD56− cells. Though with low sarcoplasmic expression, TLN1 displayed a nuclear distribution in both control and patient samples (Figure 13). To be precise, TLN1 was expressed at the nucleolus, found in the dark part of the Hoechst staining [40]. To better confirm this localization, double staining with an anti-nucleolin antibody was performed in myoblasts and myotubes, confirming the specific nucleolar distribution of TLN1 (Figure 14). Strikingly, while LGMDR1 myoblasts presented nucleolar disruption, characterized by the dispersion of nucleolar material throughout the nucleoplasm, LGMDR1 myotubes did not present such disruption (Figure 15). Finally, we also confirmed the nucleolar expression of TLN1 in muscle sections from both controls and LGMDR1 patients, with no differences (Figure 16).

### 3.5. Abnormal Nuclear Morphology in LGMDR1 Patient Samples

**Figure 17:**
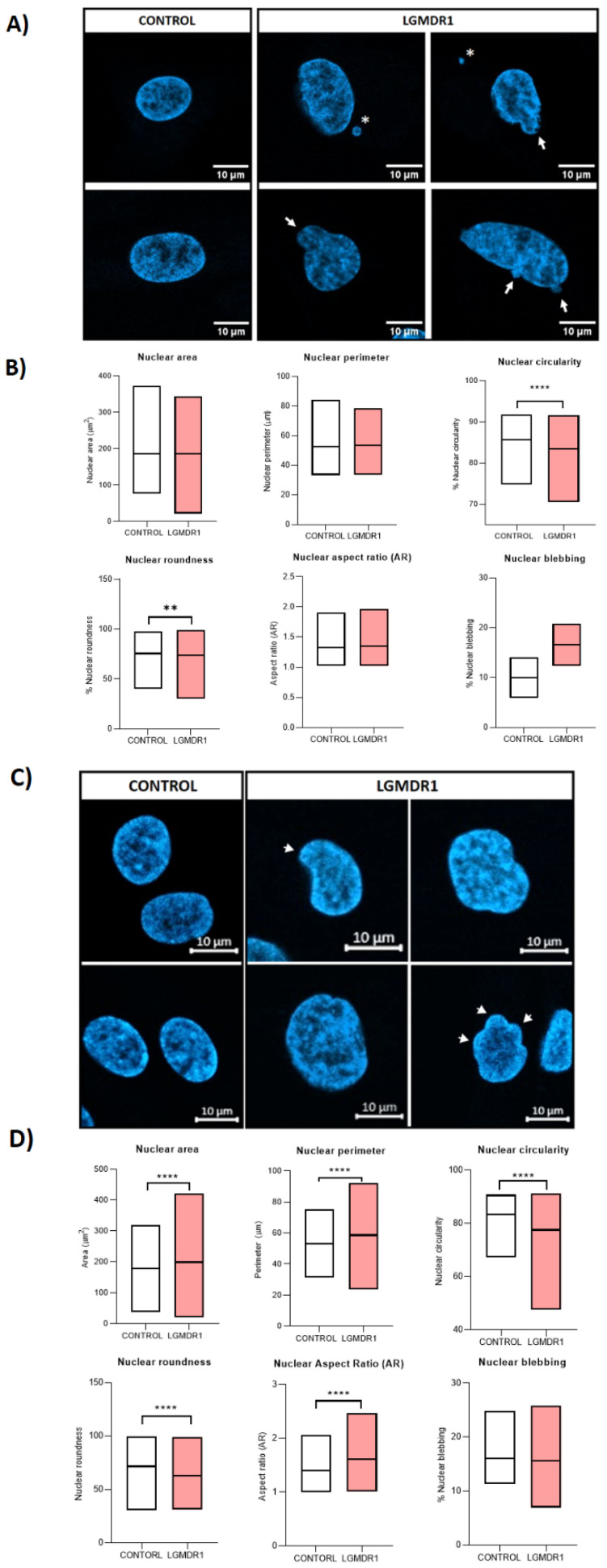

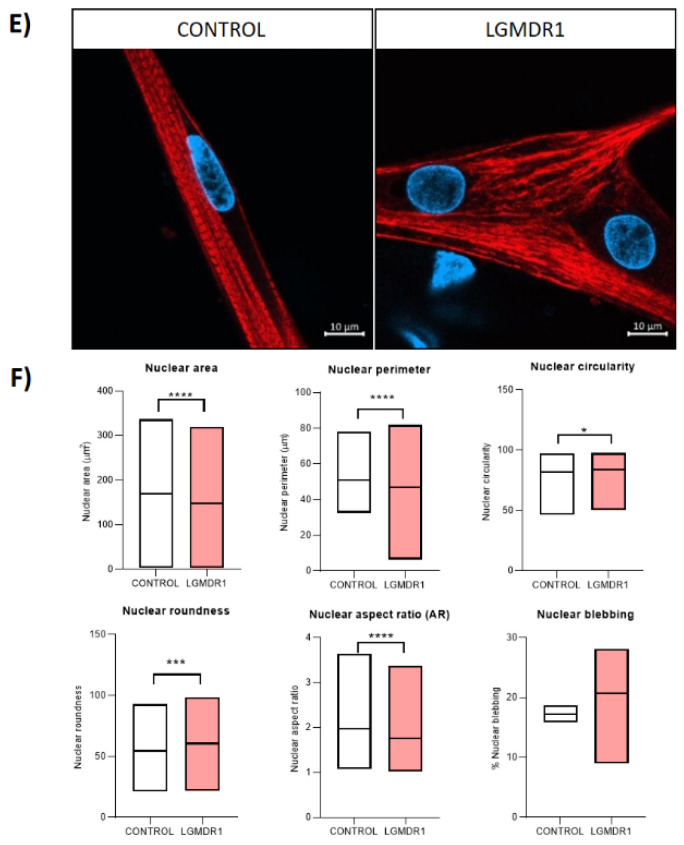
Nuclear morphology analysis in LGMDR1 cells. (**A**,**B**) CD56− cells (2 controls and 2 patients), (**C**,**D**) myoblasts (3 controls and 3 patients) and (**E**,**F**) myotubes (3 controls and 3 patients). Arrows: Nuclear blebbing. Asterisks: micronuclei. Hoechst (blue), MYHC (red). * *p* < 0.05; ** *p* < 0.01; *** *p* < 0.001; **** *p* < 0.0001. For all images, scale bars are 10 μm.

The obtained results, together with the morphological abnormalities found in myoblast nuclei from the *Capn3^KO^* mice [18], led to the morphological study of primary cells and muscle nuclei in controls and LGMDR1 patients. The nuclei of LGMDR1 patients’ CD56− cells showed significantly lower circularity and roundness (Figure 17A,B). In terms of area, perimeter and aspect ratio (AR), CD56− nuclei did not show significant differences. Despite a clear higher percentage trend of nuclear blebs in the patients’ CD56− cells, no statistical differences were observed (Figure 17A,B). Related to myoblast nuclei, LGMDR1 myoblasts showed a bigger area, perimeter and AR, but they presented lower circularity and roundness. No differences were observed in nuclear bleb percentage (Figure 17C,D). By contrast, the nuclei of LGMDR1 myotubes presented a smaller area, perimeter and AR, while circularity and roundness were higher in the patients’ myotube nuclei. Although an increasing trend was observed in the percentage nuclear blebs in LGMDR1 myotubes, it did not reach statistical significance (Figure 17E,F).

**Figure 18:**
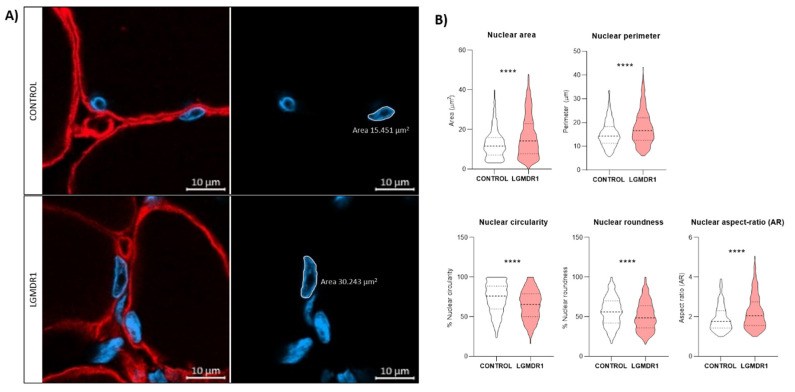
Nuclear morphology analysis in the controls and LGMDR1 patients’ muscle sections. (**A**) Confocal microscopy images of the LGMDR1 and control nuclei of muscle sections. (**B**) Nuclear morphology analysis (2 controls and 3 LGMDR1 patients). Laminin (red), Hoechst (blue). Scale bars: 10 μm. **** *p* < 0.0001.

These analyses were also performed for the muscle fiber nuclei from the controls and LGMDR1 patients in tissue sections. A bigger area, perimeter and AR were observed in the myonuclei of the LGMDR1 muscle fibers, while showing a lower circularity and roundness (Figure 18).

### 3.6. Centrosome Organization Is Impaired in LGMDR1 Cells

**Figure 19:**
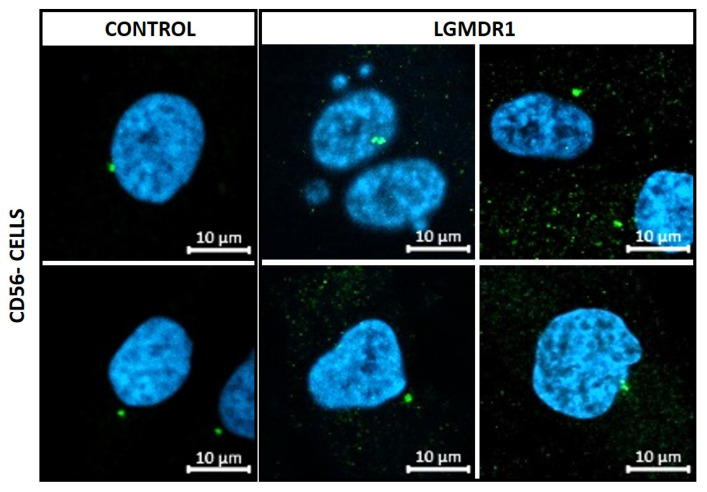
Clustered centrosomes in LGMDR1 CD56− cells (2 controls and 2 patients). Y-tubulin (green), Hoechst (blue). Scale bars are 10 μm.

Since the integrin β1 tail can regulate centrosome function [41] and centrosome reduction has been seen during myoblast differentiation [15,16,42,43], its distribution was analyzed only in the proliferating CD56− cells and myoblasts of controls and LGMDR1 patients. Clustered centrosomes were found in LGMDR1 CD56− cells (Figure 19), while centrosome aggregates were not found in the myoblasts. Since the centrosome displays a key role in the nuclear structure and its location is along the skeletal muscle cells [15,44,45], the distance between the centrosome and the nucleus was measured in myoblasts and CD56− cells.

**Figure 20:**
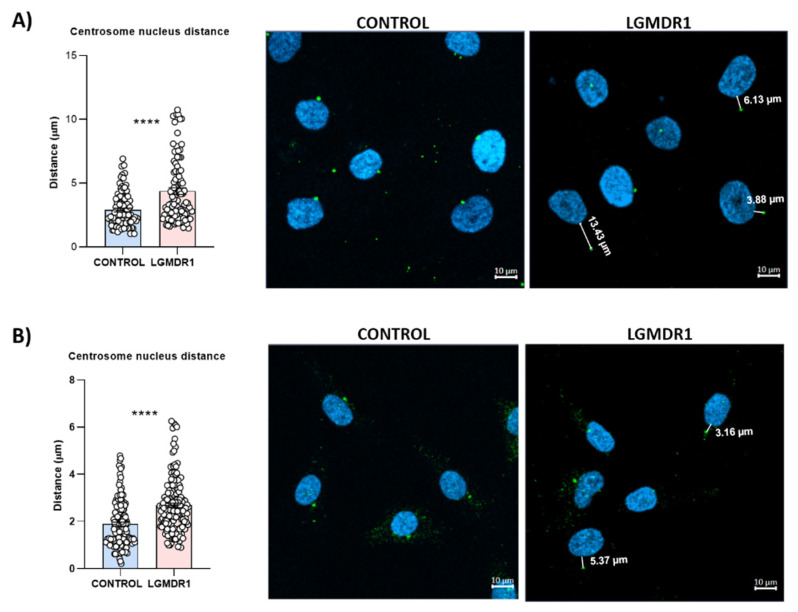
Centrosome–nucleus distance in LGMDR1 cells. Distance and representative images of (**A**) CD56− cells (2 controls and 2 patients) and (**B**) myoblasts (3 controls and 3 patients). Hoechst (blue), ϒ-tubulin (green). **** *p* < 0.0001. Scale bars: 10 µm.

**Figure 21:**
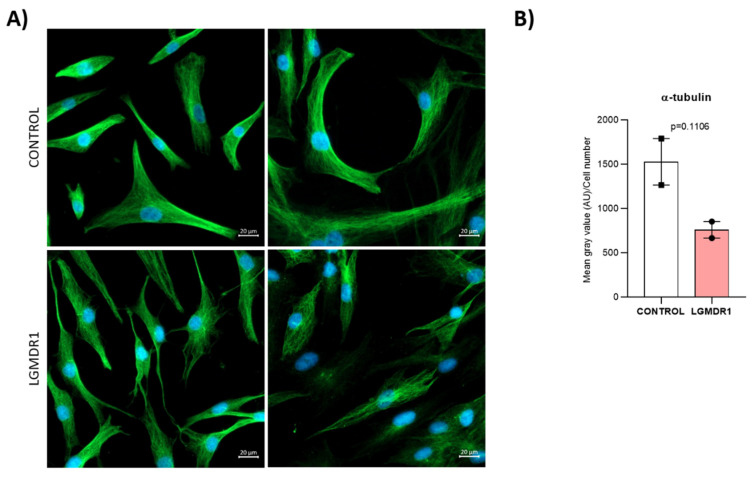
Impairment of the α-tubulin cytoskeleton in LGMDR1 in CD56− cells. (**A**) Confocal microscopy images of the disorganized α-tubulin cytoskeleton in LGMDR1 cells. α-tubulin (green), Hoechst (blue). Scale bars: 20 µm. (**B**) Quantification of the α-tubulin expression in myoblasts (2 patients and 2 controls). *p* = 0.1106.

**Figure 22:**
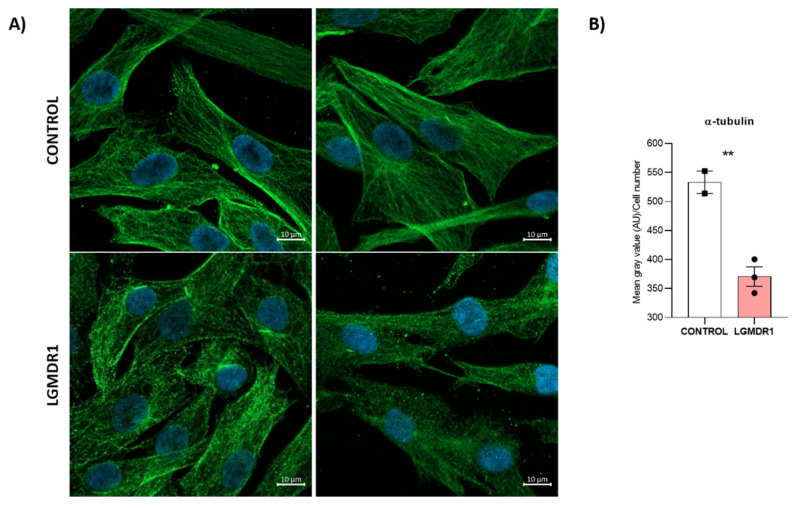
Impairment of the α-tubulin cytoskeleton in LGMDR1 myoblasts. (**A**) Representative images of the disorganized α-tubulin cytoskeleton in LGMDR1 cells. α-tubulin (green), Hoechst (blue). Scale bars: 10 µm. (**B**) Decreased α-tubulin expression in LGMDR1 myoblasts (3 patients and 2 controls). ** *p* < 0.01.

An increased distance between these two organelles was observed in LGMDR1 muscle cells: 42% and 51% higher in myoblasts and CD56− cells, respectively (Figure 20). Due to the impaired location of the centrosomes, α-tubulin immunolabelling was performed. More randomly organized microtubules and a reduced amount of protein were observed in LGMDR1 patient-derived CD56− cells and myoblasts (Figure 21 and Figure 22).

**Figure 23:**
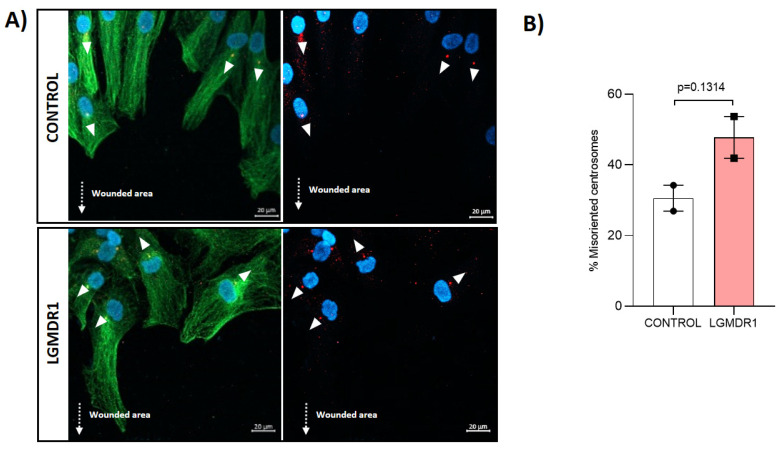
Misoriented centrosomes in CD56− cells from LGMDR1 patients after WH. (**A**) Representative images of the centrosome orientation. γ-tubulin-labelled centrosomes (red), α-tubulin-labelled cytoskeleton (green), Hoechst-labelled nuclei (blue). White arrowheads indicate the direction of the CD56− cells based on centrosome position. Punctuated arrows indicate the wounded area. Scale bars: 20 µm. (**B**) Percentage of the disoriented centrosomes in CD56− cells after wounding (2 controls and 2 patients). *p* = 0.3114.

Finally, in vitro wound-healing analysis was performed to establish whether the centrosomes showed appropriate orientations during this process. Centrosomes in LGMDR1 patients’ CD56− cells showed a disoriented localization after the wound-healing assay, although this difference was not statistically significant (Figure 23).

### 3.7. Aberrant Mitosis in LGMDR1 CD56− Cells

**Figure 24:**
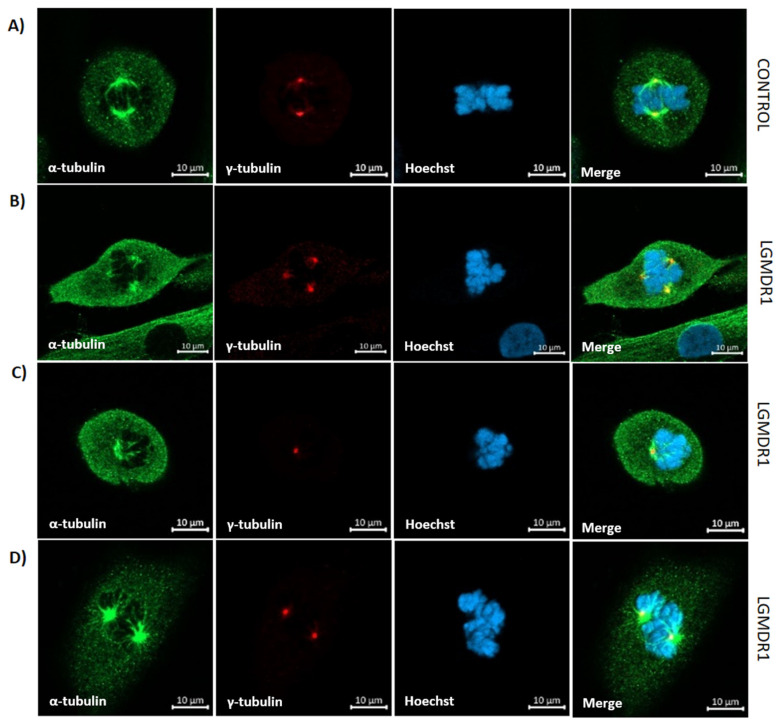
Confocal microscopy images of mitotic spindle in CD56− cells from healthy control (**A**) and LGMDR1 (**B**–**D**) (2 patients and 1 control). Normal mitotic spindle (**A**), tripolar spindle (**B**), monopolar spindle (**C**) and aberrant spindle (**D**). α-tubulin (green), γ-tubulin (red), Hoechst (blue). Scale bars: 10 µm.

Since centrosomes regulate the proper positioning of the bipolar spindle [46], mitotic divisions in the primary cells of LGMDR1 patients and controls were analyzed. Mitotic spindle abnormalities were not observed in LGMDR1 myoblasts. However, aberrant, not properly oriented mitotic spindles were found in the CD56− cells of LGMDR1 patients (Figure 24).

**Figure 25:**
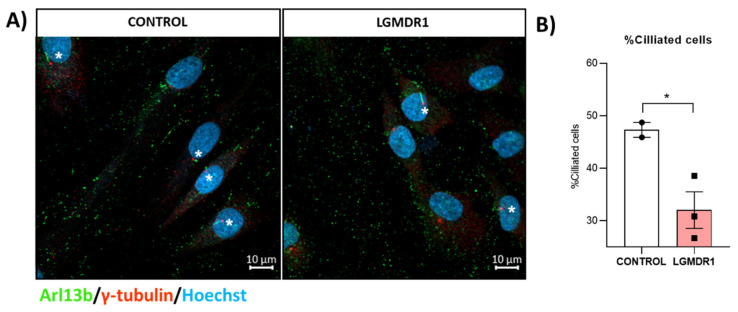
Analysis of ciliated cells in myoblasts from controls and LGMDR1 patients. (**A**) Representative images of ciliated cells (white asterisks) in control and LGMDR1 myoblasts. Arl13b (green), γ-tubulin (red), Hoechst (blue). Scale bars: 10 µm. (**B**) Ciliated cell quantification in myoblasts (two controls and three LGMDR1 patients). * *p* < 0.1.

Due to the relationship between ciliogenesis, centrosomes, and cell cycle regulation, the primary cilium was studied in LGMDR1 and control myoblasts. We found a reduction in the number of ciliated cells in LGMDR1 myoblasts compared to the controls (Figure 25).

## 4. Discussion

In this work, we analyzed the costamere protein distribution in healthy muscle and LGMDR1 patients’ muscles.

### 4.1. Fiber-Type Distribution in Healthy Muscle

The biological meaning of fiber-type specificity of the costamere proteins in muscle is not yet well established, but some studies have shown fiber-type distribution [38,47,48]. Our study revealed that ITGβ1D, TLN, ILK and FAK presented fiber-type specificity in healthy muscles, while VCL was expressed in all myofibers. However, we found some discordance with previous work regarding some of these proteins. In this study, we demonstrated that TLN1 shows predominantly a type I fiber distribution, whereas Andresen and colleagues described a lack of fiber-type specificity of this protein in human muscle [48]. Though different factors (i.e., different muscles examined) could explain this discrepancy, further analysis will be required. Conversely, while we found no fiber-type specificity for ITGβ1D, it has been reported to be predominantly expressed in mouse type II fibers [47]. Regarding these data, it is not surprising to find differences in protein distribution among human and animal muscles.

On the other hand, FAK presented a non-sarcolemmal distribution, contrary to what was observed for the rest of the proteins in healthy muscles. In patients, the expression of FAK was reduced in some type I fibers, whereas it was present in all type I fibers in the controls, suggesting that it may also affect muscle fiber development (Figure 9). Indeed, FAK, a non-receptor tyrosine kinase, activates several anti-apoptotic and cell growth pathways by translating the transduction signals carried by integrins across the cytoplasmic membrane [49].

Finally, costamere proteins were found in the microvasculature of the skeletal muscle (ITGβ1D, ILK, TLN1, FAK, VCL, and α- and β-parvins) since they are part of the dense plaques of the observed smooth muscle [50,51,52,53]. Though α- and β-parvins have been described at the sarcolemma in mouse [47] and human muscle fibers [54,55], in our study, they were only expressed in the vascular endothelium. Numerous isoforms of parvin coexist to allow for the formation of different complexes. These might impart distinct functions to each complex [56], and the isoforms we detected may only participate in blood vessels.

### 4.2. Vascular Endothelium Alteration in LGMDR1

Cells of the vascular endothelium detect changes, such as tension or pressure in blood flow, activate inflammatory pathways, recruiting leukocytes. Consequently, these cells produce or degrade the ECM and secrete cytokine/chemokines [57]. This could occur in the early stages of LGMDR1, when patients show inflammatory changes associated with the presence of eosinophils in the muscle [58], as well as the overexpression of IL-32 [7].

We observed abnormal and swollen vessel morphology in LGMDR1 muscle. ILK, TLN1, FAK, VCL, and α- and β-parvins are essential in mechanosensing and determining vasculature morphogenesis [59]. Therefore, the swollen morphology observed may have been caused by an altered distribution of these proteins in the LGMDR1 muscle vessels. In dermatomyositis, an autoimmune disease with an abnormal immune reaction to the vascular endothelium, the number of muscle capillaries per muscle fiber is reduced. Dilatation is also observed to compensate for the loss of capillaries [60,61]. This compensatory mechanism could also explain our observations in LGMDR1, although further studies are required.

### 4.3. Implications of Altered ITGβ1D Distribution in LGMDR1

Integrins are transmembrane glycoprotein receptors that regulate numerous intracellular signals and biological functions: (a) they control myoblast fusion and the migration and assembly of ECM [62,63]; (b) they are critical for changes in multiple nuclear components, nuclear regulation and epigenetic changes [64,65,66,67]; and (c) they regulate centrosome function, spindle formation and cytokinesis [41].

(a) Impaired structure and function of muscle fibers

Integrin activation is essential for normal skeletal muscle development [62,63]. The ITGβ1A isoform is replaced by the ITGβ1D isoform in muscle fiber maturation under normal physiological conditions [68]. This replacement has been shown to be altered in the myotubes of C3KO mice [69] and LGMDR1 patients’ myotubes [11].

The fact that we observed a discontinuous pattern of ITGβ1D labeling on the membrane (Figure 4) suggests that one of its main functions, assembling the ECM, is not occurring properly. ECM remodeling and inflammation are closely related in a variety of biological and pathological processes [70,71,72]. ECM remodeling is brought on by inflammation, and inflammatory processes are modulated by ECM proteins [73]. The two major receptors of fibronectin and collagen/laminin, α5β1 and α2β1, respectively, share the β1 subunit and are implicated in inflammatory signaling [73,74,75]. Thus, the observed accumulation of laminin in the LGMDR1 patient (Figure 5B) could be due to the altered distribution of ITGβ1D. Moreover, it has been reported that canonical Wnt signaling plays a fundamental role in membrane trafficking, causing the endocytosis of focal adhesion (FA) proteins and elimination of the β1 integrin from the membrane of the cell [76]. FRZB, a Wnt signaling pathway inhibitor, is overexpressed in LGMDR1 patients [11], suggesting that the deregulation of this pathway may be one of the responsible factors contributing to the observed patchy pattern of ITGβ1D in the LGMDR1 muscle fiber. Different signaling pathways may control the interactions between sarcoplasmic proteins and the sarcoplasmic tail of α or β integrins to regulate their activation [77]. TLN1 binds to the β integrin sarcoplasmic domains for activation [78,79]. The functions of CAPN3 are poorly understood. In our previous study, we conducted gene expression profiling of LGMDR1 patients’ muscles and found that TLN1 expression was not significantly altered [7]. This suggests that the observed reduction in TLN1 at the muscle sarcolemma is likely secondary to a lack of protein interaction due to integrin mislocalization. The mislocalization of integrin β1D observed in our current study may disrupt its interaction with Talin, leading to reduced Talin activation. This integrin–Talin interaction is crucial for the formation and stability of costameres, which are essential for muscle cell adhesion and signaling. While we have not conducted specific experiments to directly assess the regulation of Talin activation by CAPN3, our findings highlight the importance of integrin localization in this process. We hypothesize that CAPN3 may influence adhesion functions indirectly by affecting integrin distribution and subsequent protein interactions. We previously reported that a large proportion of genes associated with the extracellular matrix were upregulated in LGMDR1 muscles. These genes include collagen types I, III and V and cell adhesion proteins such as CD9, CD44 and fibronectin. It also identified SPARC, a matricellular glycoprotein overexpression, that may modulate cell interaction within the ECM by binding to both ECM structural components and growth factors [7]. On the other hand, when integrin binds to the ECM, Tyrosine (Tyr) phosphorylation of paxillin and FAK occurs, allowing for cell–ECM interaction [80,81]. It has been reported that β1 integrin and FAK are required for myoblast differentiation [82,83]. Furthermore, AKT, PI3K and mTOR have been documented as the downstream targets of FAK [84]. Finally, previous studies have shown that mTOR expression is also downregulated in LGMDR1 patients’ muscles [27].

Together, the observed ITGβ1D mislocalization, the lack of TLN1 in the sarcolemma and the irregular expression of FAK in LGMDR1 muscles suggest a lack of integrin activation with an altered sarcolemma, ECM assembly and signaling pathway deregulation, which may cause the frailty of the LGMDR1 muscle fibers.

(b) Nuclear alterations in muscle and cells

The arrangement of molecular complexes in the sarcoplasm and nucleus can be altered by cell surface receptors [64]. Moreover, costameres are linked to the actin cytoskeleton, and nuclear deformations can result as a consequence of intra- and extracellular forces [67]. It has been suggested that the nucleus can detect applied forces, which may regulate transcriptional activity [85,86]. There is increasing evidence that integrins are critical for changes in nuclear structure, nuclear regulation and epigenetic changes [64,65,66,67]. Therefore, the abnormal nuclear morphology observed in LGMDR1 cells may be a consequence of altered ITGβ1D distribution. Moreover, alterations in nuclear morphology are also related to chromatin rearrangement, and possibly to gene expression changes [65]. The nucleolar expression of TLN1, as well as its association with chromatin and gene expression regulation, has been described in breast epithelial cells [87]. The impaired distribution of TLN1 showed nucleolar disruption in LGMDR1 patients’ myoblasts. Nucleolar disruption is a morphological alteration of the nucleolus derived from cellular stress, presenting dispersion of the nuclear structure [88]. The inhibition of mTOR and metabolic stress are among the factors that cause nucleolar stress [89,90]. In fact, the reduced expression and phosphorylation of mTOR and altered expression of proteins involved in the metabolic process have been found in LGMDR1 muscle [27,91], suggesting a possible cause for the observed nucleolar disruption.

(c) Centrosome alterations in muscle cells

Reverte and colleagues demonstrated that the integrin β1 tail can regulate centrosome function, spindle formation and cytokinesis [41]. While microtubules control integrin activity and adhesion site remodeling, integrins stimulate microtubule nucleation, growth and stabilization [92]. Thus, abnormal integrin distribution may be the cause of the increased centrosome–nucleus distance observed in LGMDR1 patients (Figure 20). An increased centrosome–nucleus distance has also been described in fibroblasts from Duchene Muscular Dystrophy (DMD), Emery–Dreifuss, and Charcot–Marie–Tooth syndrome patients [93]. The centrosome is a complex organelle involved in microtubule nucleation and anchoring [13]. α- and β-tubulins are the major components of microtubules and γ-tubulin promotes microtubule nucleation [14,94]. Since γ-tubulin showed mislocalization in LGMDR1 cells, in consequence, α-tubulin does not polymerize properly, forming the anomalous microtubules observed in LGMDR1 cells. Moreover, it has been reported that the activation of CAPN3 results in the proteolysis of β-tubulin in mouse lenses [95], suggesting that CAPN3 may even play a role in microtubule structure organization in the muscle.

Centrosomes play a key role in chromosome instability, and they precisely maintain correct chromosome segregation during mitosis and cell polarity regulation [96]. The observed aberrant mitosis is a consequence of the altered centrosomes and microtubules structures in LGMDR1 cells. Aneuploidies and trisomies were found in the primary cells and muscles from LGMDR1 patients [29], which could be due to the observed alterations. Furthermore, the implication of the centrosomes in nuclear architecture and their function in the nuclear movement during myogenic differentiation are also known [15,44]. This could explain the clustered myonuclei observed in LGMDR1 myotubes [11].

Furthermore, centrosomes form the basal body of primary cilia, which are cellular sensory antennas that function as a hub for different signaling pathways [25], including Wnt and mTOR pathways, which are dysregulated in LGMDR1 patients [27]. The reduced ciliogenesis found in LGMDR1 myoblasts (Figure 25) may further link integrin and centrosome dysfunction with the signaling impairment previously identified in cells of this disease.

In this work, we demonstrated the importance of the correct distribution of ITGβ1D in the muscle. This was observed in the patient with the very benign phenotype (18-38110). This muscle sample presented areas of ITGβ1D and TLN1 co-localization in the sarcolemma (Figure 8), suggesting that the presence of the ITGβ1D is key to the preservation of myofiber structure and function.

In summary, ITGβ1D is a highly relevant molecule that brings together structural organization and the transmission of cellular signaling through its involvement in the composition of the costamere, ECM, and the regulation of centrosome integrity, with all of these structures being essential for the survival of the muscle fiber. Thus, muscle fiber formation in response to strain or signaling of any kind is important. It may provide a physical structure for the transmission of mechanical signals to the nucleus for the direct control of gene transcription and adaptation.

According to the involvement of ITGβ1D in a large number of cellular functions, this protein could be considered a good therapeutic target. Indeed, previous in vitro studies have shown that positive outcomes from LGMDR1 myotubes treated with either siFRZB or Tideglusib which included an increase in ITGβ1D expression [11,27], therefore suggesting that this is an affordable therapy for the preservation of muscle fiber structure.

## Figures and Tables

**Table 1:** Muscles samples from healthy controls and LGMDR1 patients for immunofluorescence analysis. Age #: Age at biopsy.

Biopsy Number	Gender	Status	Mutation 1	Mutation 2	Muscle	Age #	Age at Onset	Functional Status	Clinical Information
22-09	Male	Control	-	-	Biceps	47	-	-	-
22-18	Male	Control	-	-	Semitendinosus	24	-	-	-
18-08051	Female	Control	-	-	Quadriceps	46	-	-	-
23-10	Male	Control	-	-	Deltoid	75	-	-	-
16-6279	Male	Control	-	-	Quadriceps	35	-	-	-
19-22	Female	LGMDR1	p.(Arg788Serfs*14)	Complete deletion of *CANP3* gene	Biceps	23	23	Ambulant	Benign
21-07396	Male	LGMDR1	p.(Arg490Trp)	p.(Arg490Trp)	Tibialis anterior	46	33	Ambulant	Mild facial weakness. Proximal weakness. Bilateral scapular winging. Bilateral atrophy of biceps and pectoral muscles.
B09-83	Female	LGMDR1	DelEx2-6	DelEx2-6	Quadriceps	12	12	Ambulant	Proximal weakness. Not able to climb stairs.
18-38110	Female	LGMDR1	p.(Arg489Gln)	c.1116-2A>C	Biceps	47	Fifth decade	Ambulant	Initial symptoms were myalgia and fatigue, with persistently elevated CK, X9 (1800 UI/l). No muscle weakness at 52 years old. Unspecific slight changes in the biopsy.
97-168	Male	LGMDR1	p.(Ser479Gly)	c.1992+1G>T	N.A.	41	20	Ambulant	Muscle weakness of the pelvic and scapular girdles.

**Table 2:** Samples from healthy controls and LGMDR1 patients for cell culture.

Biopsy N.	Status	Muscle	Age	Mutation 1	Mutation 2
15-12	CONTROL	Deltoid	36	-	-
13-07	CONTROL	Deltoid	36	-	-
13-09	CONTROL	Vastus lateralis	37	-	-
22-18	CONTROL	Semitendinosus	24	-	-
23-10	CONTROL	Deltoid	75	-	-
10-39	LGMDR1	Deltoid	29	p.(Lys254del)	p.(X822Leuext62X)
09-21	LGMDR1	Biceps	19	p.(His690Argfs*9)	p.(His690Argfs*9)
09-25	LGMDR1	Deltoid	28	p.(Lys254Glu)	p.(Pro637HisfsX25)
Exp05	LGMDR1	Deltoid	13	p.(Arg788SersX14)	p.(Arg788SersX14)

## Data Availability

The original data presented in the study are openly available in ZENODO (Confocal Microscopy images-The role of Integrin B1) at https://doi.org/10.5281/zenodo.14762287, (accessed on 9 March 2025).

## Supplementary Materials

The following supporting information can be downloaded at: https://www.mdpi.com/article/10.3390/cells14060446/s1, Table S1: Primary antibodies used for immunofluorescence analysis in muscle sections and/or cells; Table S2: Fluorescent secondary antibodies used for immunofluorescence analysis.
