# Supplementary material for: The Role of Integrin β1D Mislocalization in the Pathophysiology of Calpain 3-Related Limb–Girdle Muscular Dystrophy
Source: Cells. 2025 Mar 17;14(6):446. doi: 10.3390/cells14060446 (PMC11941428; doi:10.3390/cells14060446)
Supplement: Supplementary file 1 [file cells-14-00446-s001.zip › cells-3477901-supplementary.pdf]

Table S1. Primary antibodies used for immunofluorescence analysis in muscle sections and/or cells

Antigen	Dilution	Company	Reference	Samples
ILK	1:250	Sigma-Aldrich	HPA048437	Muscle
TLN1	1:50	Cell Signaling Technology	#4021	Muscle and cells
FAK	1:50	Cell Signaling Techonology	#71433	Muscle
VCL	1:50	Cell Signaling Techonology	#13901	Muscle
α-parvin	1:50	Cell Signaling Techonology	#8190	Muscle
β-parvin	1:500	Sigma-Aldrich	HPA062601	Muscle
ITGβ1D	1:50	Merck Millipore	MAB1900	Muscle
MYHC I	1:25	DSHB	BA-D5	Muscle
Nucleolin	1:50	Thermofisher Scientific	ZN004	Muscle and cells
MYHC	1:50	DSHB	A4.1025	Cells
γ-tubulin	1:200	Sigma-Aldrich	T5326	Cells
α-tubulin	1:50	Cell Signaling Technology	#2144	Cells
Ki67	1:100	Abcam	Ab15580	Cells
Arl13b	1:100	Thermofisher Scientific	17711-1-AP	Cells

Table S2. Fluorescent secondary antibodies used for immunofluorescence analysis

Antigen	Dilution	Fluorophore conjugated	Company	Reference
Mouse	1:400	488	Thermofisher Scientific	A11001
Mouse	1:400	555	Thermofisher Scientific	A21141
Mouse-IgG2B	1:400	488	Thermofisher Scientific	A21428
Mouse-IgG1	1:400	568	Thermofisher Scientific	A21124
Mouse-IgG2A	1:400	568	Thermofisher Scientific	A21134
Rabbit	1:400	488	Thermofisher Scientific	A11034
Rabbit	1:400	555	Thermofisher Scientific	A21428
